# Modulation of doxorubicin cytotoxicity by resveratrol in a human breast cancer cell line

**DOI:** 10.1186/1475-2867-12-47

**Published:** 2012-11-16

**Authors:** Abdel-Moneim M Osman, Hadeel M Bayoumi, Sameer E Al-Harthi, Zoheir A Damanhouri, Mohamed F ElShal

**Affiliations:** 1 Department of Pharmacology, Faculty of Medicine, King Abdul-Aziz University, Jeddah, Saudi Arabia; 2 Depatment of Biochemistry, Faculty of Science, King Abdul-Aziz University, Jeddah, Saudi Arabia

**Keywords:** Doxorubicin, Resveratrol, Breast cancer cell line

## Abstract

**Background:**

Breast cancer is the most common cancer in the Arab world and it ranked first among Saudi females. Doxorubicin (DOX), an anthracycline antibiotic is one of the most effective anticancer agents used to treat breast cancer. chronic cardiotoxicity is a major limiting factor of the use of doxorubicin. Therefore, our study was designed to assess the role of a natural product resveratrol (RSVL) on sensitization of human breast cancer cells (MCF-7) to the action of DOX in an attempt to minimize doxorubicin effective dose and thereby its side effects.

**Methods:**

Human breast cancer cell line MCF-7, was used in this study. Cytotoxic activity of DOX was determined using (sulforhodamine) SRB method. Apoptotic cells were quantified after treatment by annexin V-FITC- propidium iodide (PI) double staining using flow-cytometer. Cell cycle disturbance and doxorubicin uptake were determined after RSVL or DOX treatment.

**Results:**

Treatment of MCF-7 cells with 15 μg/ml RSVL either simultaneously or 24 h before DOX increased the cytotoxicity of DOX, with IC50 were 0.056 and 0.035 μg/ml, respectively compared to DOX alone IC50 (0.417 μg/ml). Moreover, flow cytometric analysis of the MCF-7 cells treated simultaneously with DOX (0.5 μg/ml) and RSVL showed enhanced arrest of the cells in G_0_ (80%). On the other hand, when RSVL is given 24 h before DOX although there was more increased in the cytotoxic effect of DOX against the growth of the cells, however, there was decreased in percentage arrest of cells in G_0_, less inhibition of DOX-induced apoptosis and reduced DOX cellular uptake into the cells.

**Conclusion:**

RSVL treatment increased the cytotoxic activity of DOX against the growth of human breast cancer cells when given either simultaneously or 24 h before DOX.

## Introduction

Breast cancer is the leading cause of death in women worldwide and it is the most common cancer in the Arab world. It affects women at an early age compared with women in western countries [1]. Doxorubicin (DOX), an anthracycline antibiotic is among the most effective anticancer agents used to treat breast cancer [2]. It exerts its cytotoxic effect by intercalating between DNA base pairs on the double helix and inhibiting topoisomerase II (TOPO-II), the enzyme responsible for DNA helix conformation and stability. Unfortunately, chronic cardiotoxicity including development of a cardiomyopathy is a major limiting factor of the chemotherapeutic use of doxorubicin [3]. In an attempt to minimize DOX effective chemotherapeutic dose and thereby its side effects, a variety of approaches have been Investigated. One of them is the search for natural compounds with chemopreventive or anticancer properties that can be used in combination with doxorubicin. Resveratrol (RSVL) (trans – 3, 5, 4 – trihydroxystilbene) is a naturaly occurring poly-phenolic compound found primarily in root extracts of the oriental plant Polygonum cuspidatum and many other plant species [4]. It is highly abundant in skins of red grapes and moderately abundant in peanuts and blueberries [4]. It has recently been discovered that it has many beneficial effects in different biological systems, which include anti-inflammatory, antioxidant, anti-neoplastic, anti-carcinogenic, anti-tumorigenic, cardioprotective, neuroprotective, anti-aging and antiviral effects [4]. Its potential chemopreventive and chemotherapeutic activities have been demonstrated in all three stages of carcinogenesis (initiation, promotion, and progression) [5]. Resveratrol exhibits anticancer properties in a wide variety of tumor cells, including breast cancer cells [6]. The growth-inhibitory effect of RSVL is mediated through different mechanisms [7]. Therefore this study was aimed to explore whether the natural product resveratrol could enhance the cytotoxic effect of DOX against the growth of human breast cancer cell line (MCF-7 cell line). We investigated the possible mechanisms of interaction between DOX and RSVL regarding DOX cytotoxicity, apoptosis induction, cellular uptake and cell cycle progression of breast cancer cells in presence and absence of RSVL.

## Materials and methods

### Drugs and chemicals

DOX hydrochloride and RSVL were purchased from Sigma Aldrich (St. Louis, Mo, USA). The stock solutions of both drugs were dissolved in phosphate buffered saline (PBS) and preserved at –20°C. The solution was diluted in Dullbecco’s modified Eagles medium (DMEM) or PBS immediately before each experiment to the desired final concentrations. Dullbecco’s modified eagles medium (DMEM), Trypsin/EDTA, Phosphate buffered saline (PBS), Penicillin G and Steptomycin antibiotics, Acetic acid, Trizma base, SulphoRhodamine- B (SRB), Propidium Iodide (PI) and Annexin V-FITC apoptosis detection kit were purchased from Sigma Aldrich Co.

### Cells and cell cultures

Human breast cancer cell line MCF-7, was used in this study. It was obtained from National Cancer Institute, Cairo University, Egypt.

The adherent cells were grown as “monolayer culture” in DMEM supplemented with Penicillin (100 IU/ml), Streptomycin (100 μg/ml) and 10% Fetal bovine serum. Cells were cultured at 37°C in a humidified 5% CO_2_ atmosphere and were passaged every 4–5 days.

## Methods

### Assessment of cytotoxic activity

Cytotoxicity was determined using (sulforhodamine) SRB method as previously described by Skehan *et al.*[8]. Cells were seeded in 96 well microtiter plates at a concentration of 30 × cells/well in DMEM supplemented medium. After 24 h, cells were incubated for additional 48 h with various concentrations of DOX and RSVL in the following ranges: 0.0312–5 μg/ml for DOX and 15 μg/ml for RSVL. Drugs were added either in a simultaneous or sequential manner. In sequential treatment, the cells were pretreated with RSVL for 24 h, and then followed by DOX for further 48 h**.** Cells were fixed in situ by adding 50 μL of cold 50% TCA for 1 h at 4°C. the supernatant is then discarded, and the wells were washed five times with distilled water, air dried, stained for 30 min at room temperature with 0.4% SRB dissolved in 1% acetic acid and then washed four times with 1% acetic acid. The plates were air dried and the dye was solubilized with 100 μl/well of 10 mM Tris base (PH 10.5) for 10 min. The optical density (OD) was obtained using ELx808 Absorbance Microplate Reader obtained from BioTek Instruments,Inc (Winooski,VT, U.S.A.) at wavelength of (490–530 nm).

$$Surviving\;fraction=\frac{Optical\;density\;oftreated\;cells\;}{Optical\;density\;of\;untreated\;control\;cells}$$

**IC50** (the concentration of DOX necessary to produce 50% inhibition of cell growth) was calculated from linear equation of the survival fraction curve.

Y=m X+b

Where:

**Y** = 0.5 (the surviving fraction when there is a 50% inhibition of cell growth).

**m** = the slope.

**X** = dose of DOX induces 50% inhibition.

**b** = the y-intercept.

### Flow-cytometric assay of apoptosis

Apoptotic cells were quantified by Annexin V-FITC- Propidium iodide (PI) double staining, using an Annexin V-FITC apoptosis detection kit according to the method of Van Engeland *et al.*[9]. Cells were seeded in 12-well plates at cell density of 6–8 × cells/well in DMEM supplemented medium. Twenty four hours later, cells were incubated for additional 48 h with 15 μg/ml RSVL and various concentrations of DOX in the following range: 0.25–0.5 μg/ml. Drugs were added in a simultaneous or sequential manner. In sequential treatment, the cells were pretreated with RSVL for 24 h, and then followed by DOX for additional 48 h**.** Cell medium was then removed and the wells were washed with PBS, then the cells were harvested with trypsin/EDTA. Cells were washed once with PBS following trypsinization, resuspended in 1 ml of Binding Buffer. Annexin V FITC Conjugate were added to the cells according to manufacturer’s instructions for 10 min at room temperature while protected from light. Fluorescence of the cells was read immediately by flow cytometer (NAVIOS Beckman Coulter, U.S.A.).

### Cell cycle analysis

Cells were plated in 12-well plates at cell density of 6–8×10^5^ cells/well in DMEM supplemented medium. Twenty four hours later, cells were incubated for additional 48 h with 15 μg/ml RSVL and a various concentrations of DOX in the following range: 0.125–0.5 μg/. Drugs were added in a simultaneous or sequential manner. In sequential treatment, the cells were pretreated with RSVL for 24 h, and then followed by DOX for 48 h**.** Cell medium was then removed and the wells were washed once with PBS. Cell cycle analysis was performed according to the method of Pozarowski and Darzynkiewicz, [10].

The cells were harvested with trypsin/EDTA, washed once with PBS and then resuspended in 0.5 ml of 0.05% Triton X-100 for 10 min at room temperature. Staining of cellular DNA was performed by adding 1 ml of 50 μg/mL PI to each cell suspension for 20 min at room temperature. Cell cycle analysis was performed by using flow cytometer (Becton Dicknoson (BD) FACSCalbur, USA)**.**

### Assessment of doxorubicin cellular accumulation

DOX cellular accumulation assessment in MCF-7 cells was performed using spectrofluorometer (F-2000 Fluorescence spectrophotometer Hitachi, Japan) according to the method of Kitagawa *et al.*[11]*.* DOX fluorescence intensity was measured at excitation and emission wavelengths of λ ex = 496 nm and λ em = 592 nm, respectively to determine DOX concentration.

$$\text{DOX cellular accumulation ratio}=\frac{\text{DOX concentration in RSVL treated cells}}{\text{DOX concentration in cells treated with DOX alone}}$$

### Statistical analysis

Statistical analysis was performed using SPSS (statistical package of social sciences, version 16). One way analysis of variance (ANOVA) followed by least significant difference (LSD) for post hoc analysis, was used for multiple comparisons. Statistical significance was acceptable to a level of p < 0.05.

## Results

### Effect of RSVL treatment on the cytotoxic activity of DOX

Cytotoxicity was expressed as the percentage of surviving fraction compared with untreated control cells (Tables 1 and 2). Treatment with DOX alone showed IC50 (the concentration necessary to produce 50% inhibition of cell growth) value of 0.417 μg/ml. Simultaneous addition of 15 μg/ml RSVL with or 24 h before DOX was found to sensitize MCF-7 cells to the cytotoxic effect of DOX., IC50 were 0.056 μg/ml and 0.035 μg/ml, respectively, which were significantly different from DOX alone. At the same time RSVL 24 before DOX showed IC50 value significantly different from DOX+RESVL supplied simultaneously.

**Table 1:** Effect of DOX and RSVL (15 ug/ml) on the growth of MCF-7 cells

	**Surviving Fraction**		
**DOX Concentration (μg/ml)**	**DOX**	**DOX+RSVL Simultaneously**	**DOX+RSVLRSVL 24h before**
**0.0625**	0.97 ± 0.17	0.10^**a**^ ± 0.013	0.173^a^ ± 0.03
**0.25**	0.92 ± 0.112	0.091^a^ ± 0.018	0.124^**a**^ ± 0.006
**0.5**	0.29 ± 0.061	0.089^**a**^ ± 0.034	0.047^a,b^± 0.003

Each data is the mean ± S.E.M of two experiments each one in duplicate. ^a^Significantly different from DOX at P-value < 0.05.

^**b**^ Significantly different from simultaneous DOX+RSVL at P-value < 0.01.

**Table 2:** Effect of DOX and/or RSVL on the growth of MCF-7 cells

**Treatment**	**IC50 (μg/ml)**
**DOX**	0.417 ± 0.107
**DOX + RSVL (15 μg/ml) (supplied simultaneously)**	0.056^**a**^ ± 0.026
**DOX + RSVL (15 μg/ml) (RSVL supplied 24 h before DOX)**	0.035^**a,b**^ ± 0.016

IC50: the concentration of DOX necessary to produce 50% inhibition of cell growth.

^**a**^ Significantly different from DOX at P-value < 0.05.

^b^ Significantly different from DOX+ RSVL (Simultaneously) at P-value < 0.05.

### Effect of RSVL and DOX treatment on apoptosis induction

**Figure 1:**
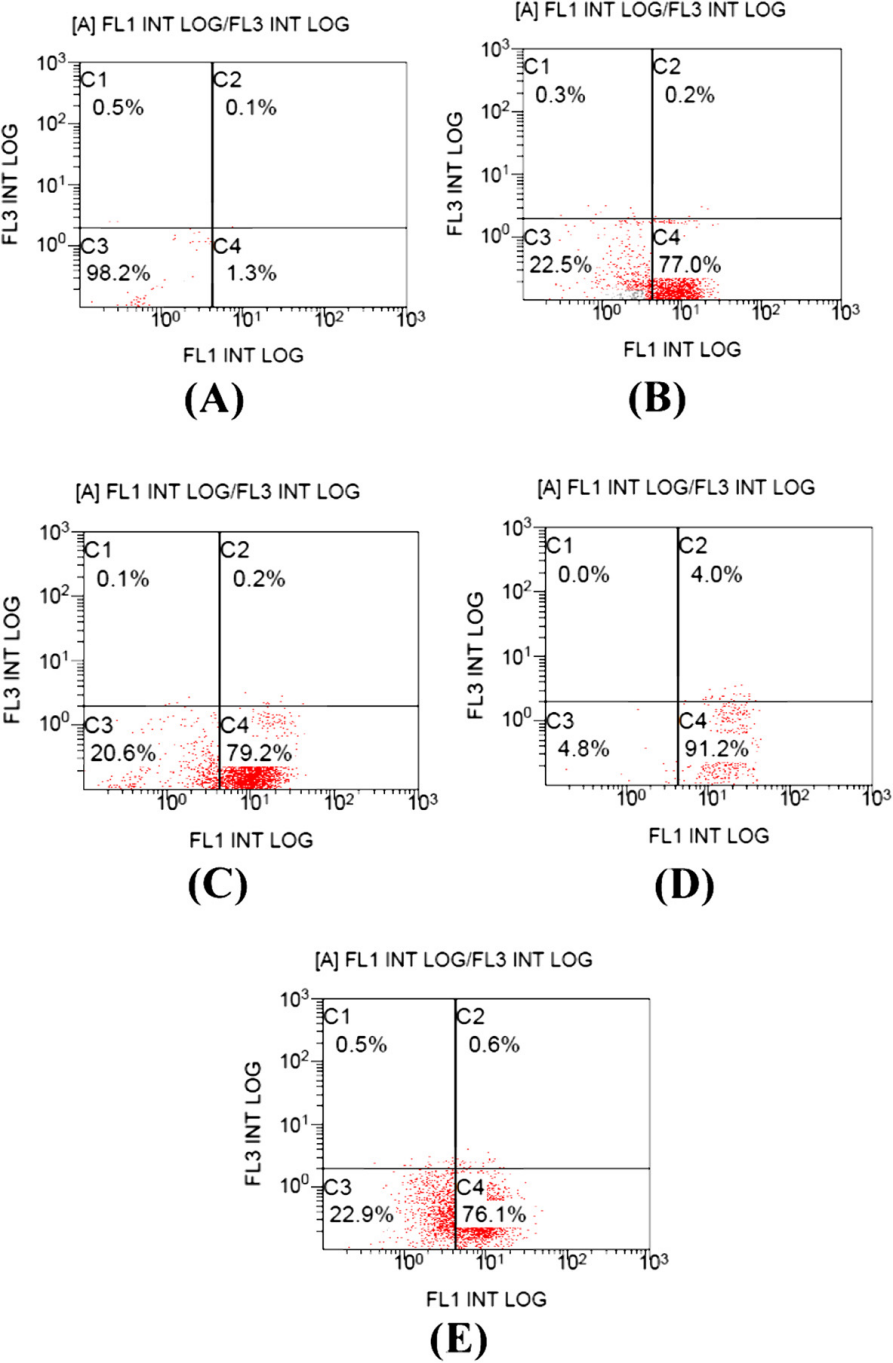
**Effect of DOX and/or RSVL on apoptosis induction in MCF-7 cells.** Apoptosis was analyzed after 48 h of exposure to drugs by staining with propidium iodide (PI, y-axis) and annexin- FITC (x-axis). (**A**) control, (**B**) cells treated with 15 μg/ml RSVL, (**C**) cells treated with 0.25 μg/ml DOX, (**D**) cells treated with 0.25 μg/ml DOX and RSVL 15 μg/ml in simultaneous manner, (**E**) cells treated with 0.25 μg/ml DOX and RSVL 15 μg/ml given 24 h before DOX. The percentage of cells in each quadrant are indicated (C1: necrosis, C2: late apoptosis, C3: live cells, C4: early apoptosis). The experiment was repeated twice each one in duplicate.

**Figure 2:**
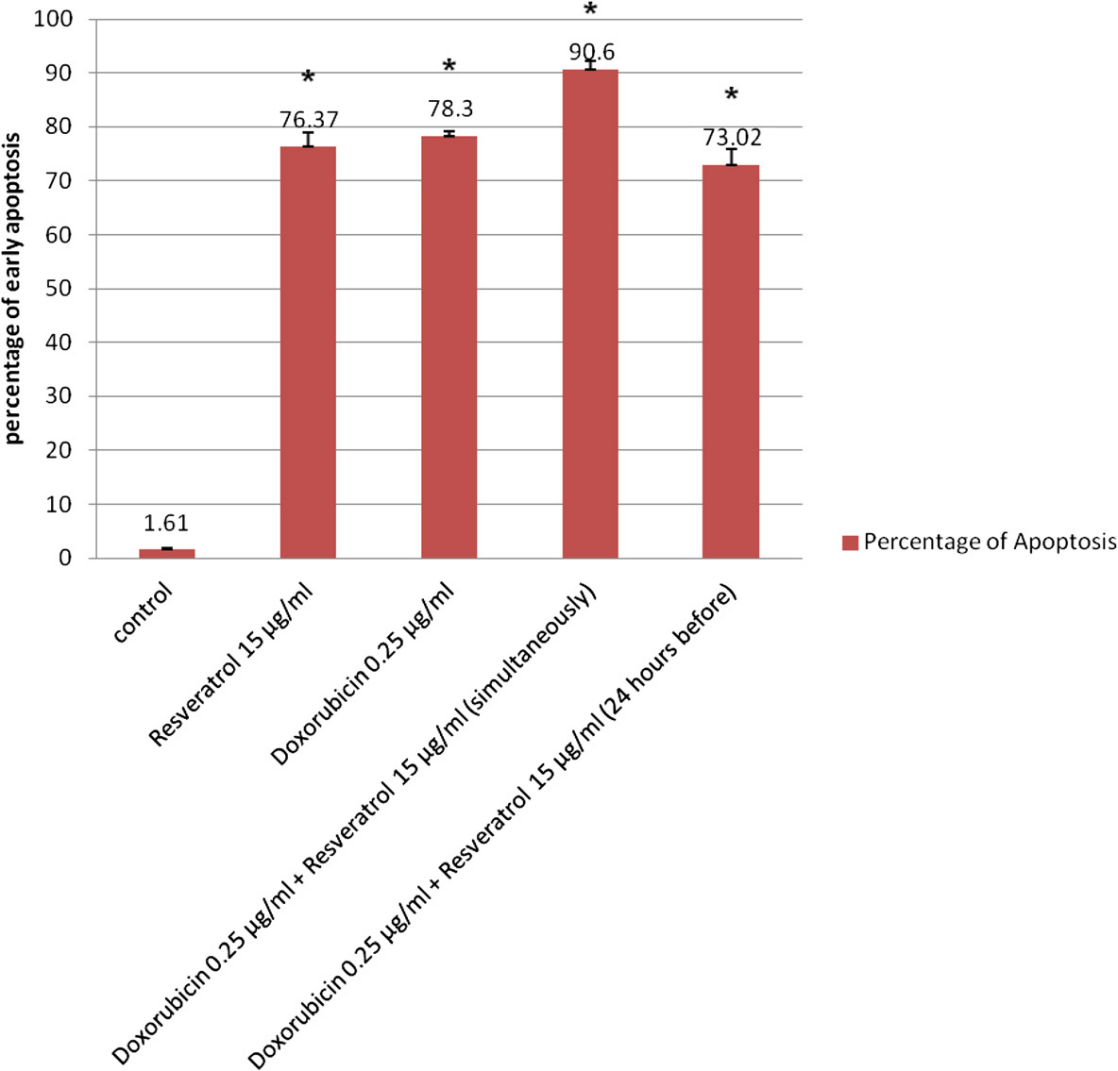
**Effect of 0.25 μg/ml DOX and/or RSVL on apoptosis induction in MCF-7 cells.** Apoptosis was analyzed after 48 h of exposure to drugs. Each point is the mean ± S.E.M of two experiments each one in duplicate. * Significantly different from control at P-value < 0.05.

Apoptosis was determined by flow cytometry in MCF-7 cells that have been stained with FITC-annexin V and PI. Percentages of cells in each quadrant in Figures 1 and 2 are representative of: (C1) necrosis, (C2) late apoptosis, (C3) live cells, and (C4) early apoptosis. Figure 1 shows control MCF-7 cells (A), cells treated with 15 μg/ml RSVL (B) and cells treated with 0.5 μg/ml DOX alone (C) or in the presence of 15 μg/ml RSVL given simultaneously with 0.5 μg/ml DOX (D) or 24 h before it (E). Figure 2 showed cells treated with 0.25 μg/ml DOX alone (F) or in the presence of 15 μg/ml RSVL given simultaneously with 0.25 μg/ml DOX (G) or 24 h before it (H).

**Figure 3:**
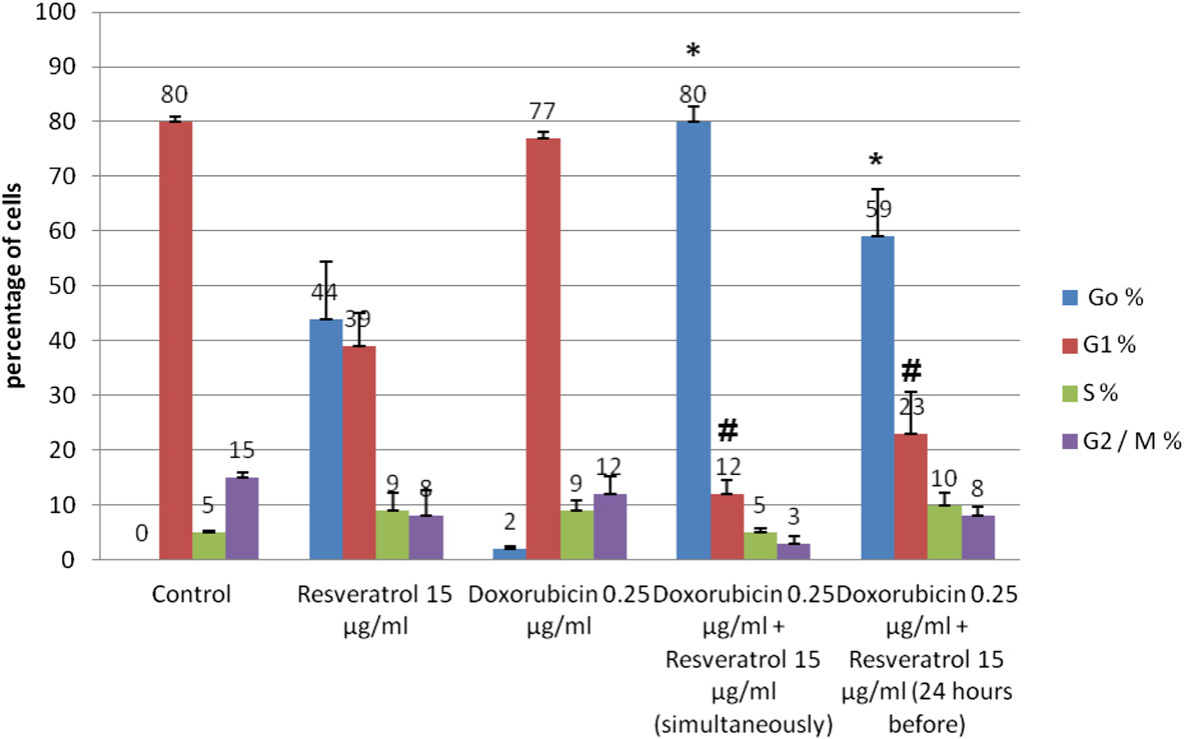
**Effect of 0.25 μg/ml DOX and/or RSVL on cell cycle phase distribution of MCF-7 cells.** Cell cycle distribution was analyzed after 48 h of exposure to drugs by staining with propidium iodide (PI). Each point is the mean ± S.E.M of two experiments each one in duplicate. * Significantly different from the corresponding DOX-induced Go arrest at P-value < 0.05. **#** Significantly different from the corresponding DOX-induced G1 arrest at P-value < 0.05.

The percentage of early apoptotic cells (Annexin V-positive cells) were dramatically increased after treatment with DOX or DOX + RSVL in comparison to the control cells (1.3% early apoptotic cells). Treatment with 0.25 μg/ml DOX showed 76.1% of early apoptotic cells. While combination treatment of 0.25 μg/ml DOX with 15 μg/ml RSVL simultaneously or RSVL 24 h before DOX showed 91.2%, and 76.1% of early apoptotic cells, respectively (Figure 3).

### Effect of RSVL and/or DOX treatment on cycle phase progression of MCF-7

**Figure 4:**
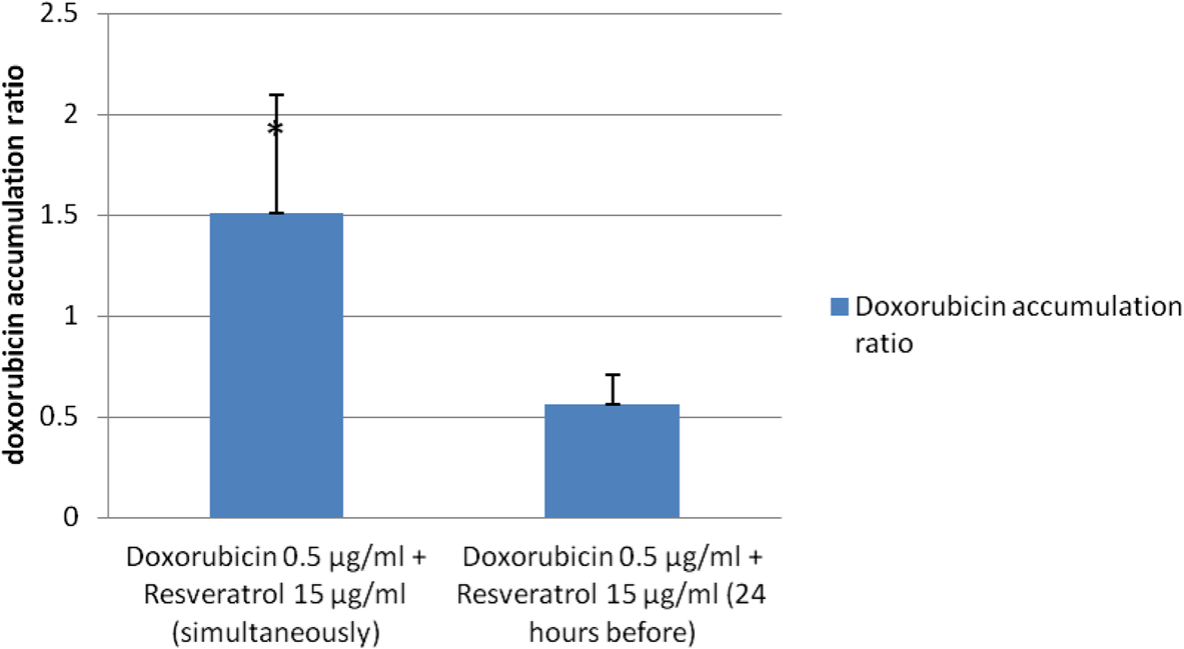
**Effect of RSVL supplied either simultaneously or 24 h before 0.5 μg/ml DOX on DOX cellular uptake in MCF-7 cells.** DOX accumulation ratio was calculated after 48 h of exposure to drugs as seen in materials and methods (3.2.4.3). Each data is the mean ± S.E.M of two experiments each one in duplicate. * Significantly different from DOX+RSVL (24 h before) at P-value < 0.05.

Treatment with different concentrations of DOX (0.125, 0.25 and 0.5 μg/ml), showed a preferential block of MCF-7 cells in S phase (data not shown). DOX concentration increased cell accumulation in S phase to 8.41% and 10.9% at dose level of 0.25 and 0.5 ug/ml,respectively (Figure 3) compared with cells in G_1_ phase. Treatment with 15 μg/ml RSVL showed arrest of cells in G_0_ and S phases compared with G_1_ phase cells 44.53% and 8.82%, respectively (Figure 4).

Combination treatment of DOX 0.25 μg/ml with 15 μg/ml RSVL simultaneously showed a huge increase in the percentages of cells in G_0_ phase in comparison with G_1_ phase cells. The cell accumulation percentage at G_0_ phase was 79.77% when treated with 0.25 μg/ml DOX given simultaneously with 15 μg/ml RSVL (Figure 4). Combination treatment of the same concentration of DOX (0.25 μg/ml) with 15 μg/ml RSVL 24 h before DOX also showed an increase in the percentages of cells in G_0_ phase compared with G_1_ phase cells but the increase was less than that observed after the simultaneous trearment. The cell accumulation percentages at G_0_ phase were 58.61% for cells treated with 15 μg/ml of RSVL followed by 0.25 μg/ml DOX after 24 h, (Figure 4).

### Effect of RSVL on doxorubicin cellular accumulation

MCF-7 cells were treated with different concentrations of DOX (0.125, 0.25 and 0.5 μg/ml) in the presence or absence of 15 μg/ml RSVL given simultaneously or 24 h before DOX.

Table 3 showed DOX cellular uptake concentrations after treatment with DOX alone, DOX+RSVL (simultaneously) and DOX+RSVL (RSVL supplied 24 h before DOX). DOX cellular uptake concentrations were 0.022, 0.027 and 0.041 μg/6 × cells in MCF-7 cells treated with 0.125, 0.25 and 0.5 μg/ml DOX, respectively.

**Table 3:** Effect of RSVL treatment on the cellular uptake of DOX in MCF-7 cells

**Treatment**	**DOX Concentration (μg/106 x cells)**		
	**Alone**	**Simultaneously with RSVL**	**RSVL 24 h before**
**DOX (0.5 μg/ml)**	0.041 ± 0.008	0.062 ^**a**^ ± 0.011	0.023 ± 0.001
**DOX (0.25 μg/ml)**	0.027 ± 0.004	0.031 ± 0.005	0.024 ± 0.001
**DOX (0.125 μg/ml)**	0.022 ± 0.004	0.021 ± 0.004	0.013 ± 0.002

Each data is the mean ± S.E.M of two experiments each one in duplicate. ^a^Significantly different from DOX at P-value < 0.05.

Table 3 and Figure 4 showed that RSVL treatment simultaneously with DOX increased its cellular accumulation gradually. The accumulation ratio was 1.58 when cells treated with 0.5 μg/ml DOX simultaneously with 15 μg/ml RSVL, respectively.

Contrary to the above results, in MCF-7 cells that were pre-treated with 15 μg/ml RSVL 24 h before the cellular accumulation ratio was 0.58 compared with 1.58 when cells treated with 0.5 μg/ml DOX after 24 h of treatment with 15 μg/ml (Figure 4).

## Discussion

Doxorubicin is the most widely used drug in the treatment of a variety of human neoplasms, However, with the increasing use of DOX, acute as well as chronic cumulative dose-dependent cardiomyopathy has been recognized as the major limiting factor for DOX chemotherapy [12,13]. Therefore, in this study we investigated the modulatory effect of the natural polyphenolic compound, RSVL on DOX cytotoxicity in MCF-7 human breast cancer cell line.

Treatment of MCF-7 cells with different DOX doses alone was observed to be cytotoxic to the cells. The cytotoxicity of DOX has been confirmed by the results of induction of apoptosis and cell cycle progression, where 0.25 μg/ml DOX induced 49 –fold increase in early apoptotis and 2-fold increase in arrest of the cells in S phase in comparison with control cells.

Similar results was obtained following single treatment of DOX in MCF-7 cells [14]. In support of the importance of cell-cycle arrest to DOX cytotoxicity, it has been found that P388 leukemia cells synchronized in S and G_2_/M phases were more sensitive to DOX than cells in G_1_ phase [15]. Our results, have further confirmed the fact that anthracyclines are mostly active on proliferating cells in S and G_2_/M phases due to the maximal expression of their target enzyme TOPO II at these phases [16,17].

Resveratrol is known to have both cardioprotective and antitumor activities [7,18] and it can attenuate DOX-induced early cellular damage in cancer patients [19]. Thus RSVL is a perfect candidate to be used as a sensitizing agent to modulate the cytotoxic effect of DOX against the growth of breast cancer cells. We also observed that, MCF-7 cells treated with RSVL alone showed high increase in early apoptosis, S-phase and in G_0_ phase (Figures 2 and 3). Resveratrol has previously been shown to induce dose-dependent cell cycle arrest, growth inhibition or apoptosis in several human cancer cell lines [20]. Resveratrol apoptosis induction effect in tumor cell line from different origins was shown to be through a lot of different regulatory mechanisms [21,22]. Previous studies on the effects of RSVL on the cell cycle of many cell lines including MCF-7 cells, demonstrated the ability of RSVL to block the S–G_2_ transition resulting in a concentration-dependent accumulation of cells in S or G_1_ phase which may be due to inhibition of the enzymes used for DNA replication such as ribonucleotide reductase [20,23-25]. Other mechanisms that could explain RSVL-induced S phase arrest is the increase expression of p53, a tumor suppressor protein [26], the increase expression of positive G_1_/S regulators, such as cyclin D1 and cyclin E which are responsible for S phase entry [27], depletion of survivin, an inhibitor of apoptosis protein [7]. Resveratrol-induced S phase arrest would eventually lead to apoptotic death as indicated by the very high increase in G_0_ phase arrest (Figure 3).

Treatment with 15 μg/ml RSVL supplied simultaneously with different DOX concentrations enhanced the cytotoxic effect of DOX significantly. There was a 7.4-fold decrease in IC50 in cells treated with DOX and RSVL simultaneously as compared with DOX treated cells (Table 2). To gain further insight into the interaction mechanisms between DOX and RSVL, apoptosis assay, flow cytometric DNA analysis and DOX cellular uptake assay were performed. Apoptosis assay showed a small increase of the early apoptotic cell percentages in the simultaneous treated cells as compared with DOX treated group. The smaller DOX dose used simultaneously with RSVL showed a stronger increase in apoptosis as compared with DOX treated group (Figure 2). Furthermore, flow cytometric analysis revealed that simultaneous treatment of DOX with RSVL induced preferential cell arrest at G_0_, there were 41-fold increase in percentages of G_0_ phase arrest for treated cells (Figure 3). Several studies have reported that RSVL molecular mechanisms of sensitization for drug induced apoptosis involved cell cycle arrest in S phase [27,28], which has been used as a strategy to increase drug incorporation into cells. Thus, the cooperative effect of RSVL and the cell cycle-dependent drug DOX may result from RSVL-induced cell cycle arrest in S phase, thereby exposing a higher proportion of tumor cell population to DOX, therefore, more cells will undergo apoptosis and leave the cycle to enter the apoptotic G_0_ phase.

These findings have been further confirmed by the observed increased in DOX cellular uptake after the simultaneous treatment with RSVL, which was in a dose dependent manner. There were an increase in DOX accumulation ratios for cells treated with DOX and RSVL, (Figure 4). This implies that, RSVL not only exposed higher proportion of MCF-7 cells to DOX by inducing cell cycle arrest in S phase but it also increased the DOX concentration available inside the cells. The increase in DOX cellular uptake inside the MCF-7 cells may be explained based on the inhibition of P-glycoprotein and multidrug resistance (MDR) [29] that plays very important role in the absorption, distribution, and elimination of DOX, and thus determines its efficacy and toxicity [29,30]. Surprisingly our results showed that when RSVL was given prior to DOX, although it was more cytotoxic against the growth of MCF-7 cells, we noticed slight inhibition of DOX-induced apoptosis, less percentage of cells arrest in G_0_ and decreased DOX cellular uptake into the cells compared with simultaneous treatment with DOX and RSVL.

The decrease of DOX cellular uptake in MCF-7 cells and the arrest of cells in S phase suggest that the enhanced growth inhibitory effects observed after the sequential RSVL and DOX treatment may not be caused by the synergism between DOX and RSVL or by the increased DOX cellular uptake, but this may be caused by the cytotoxic activity of RSVL itself [20,27].

Recently (2012), RSVL was found to reduce the intracellular accumulation of rhodamine 123 in colon cancer cell line suggesting that RSVL enhances the activity of P-glycoprotein [31]. These conflicting findings could be explained on the following basis: MDR can be acquired after initial exposure to the anticancer drugs [32]. In addition several studies have found that some of the well known P-glycoprotein antagonists such as verapamil and cyclosporine A can induce P-glycoprotein expression in colon carcinoma cells [33]. It is important to note that the time needed for expression and inhibition of P-glycoprotein by their antagonists is controversial. Therefore, based on our results we can say that RSVL antagonizes or inhibits P-glycoprotein when it is given simultaneously with DOX thereby causing an increase in DOX cellular uptake [29]. However, when it is given 24 h before DOX it enhances the P-glycoprotein expression. The 24 h period between RSVL and DOX is considered as an intial exposure that will enhance the expression of P glycoprotein and thereby MDR that will lead to the decrease in DOX cellular uptake. Further studies are needed to investigate how different sequence of treatment of RSVL and DOX could affect the P-glycoprotein activity and hence by the DOX intracellular accumulation in MCF-7 cells.

## Competing interests

The authors declare that they have no competing interests.

## Authors’ contribution

Abdel-Moneim, Zohir, sameer and Hadeel sharing in experimental work and writing the manuscript Mohamed Elshal did the flow cytometric analysis. All authors read and approved the final manuscript.
